# Dietary Nucleotides Supplementation Improves the Intestinal Development and Immune Function of Neonates with Intra-Uterine Growth Restriction in a Pig Model

**DOI:** 10.1371/journal.pone.0157314

**Published:** 2016-06-15

**Authors:** Lianqiang Che, Liang Hu, Yan Liu, Chuan Yan, Xie Peng, Qin Xu, Ru Wang, Yuanfang Cheng, Hong Chen, Zhengfeng Fang, Yan Lin, Shengyu Xu, Bin Feng, Daiwen Chen, De Wu

**Affiliations:** 1 Institute of Animal Nutrition,Sichuan Agricultural University, No. 211, Huimin Road, Wenjiang District, Chengdu, Sichuan, 611130, People’s Republic of China; 2 College of Food Science, Sichuan Agricultural University, No. 211, Huimin Road, Wenjiang District, Chengdu, Sichuan, 611130, People’s Republic of China; 3 Institute of livestock nutrition, Tongwei group Co., Ltd., No. 588, Tianfu Road, Gaoxin District, Chengdu, Sichuan, 610041, People’s Republic of China; Centre Hospitalier Universitaire Vaudois, FRANCE

## Abstract

The current study aimed to determine whether dietary nucleotides supplementation could improve growth performance, intestinal development and immune function of intra-uterine growth restricted (IUGR) neonate using pig as animal model. A total of 14 pairs of normal birth weight (NBW) and IUGR piglets (7 days old) were randomly assigned to receive a milk-based control diet (CON diet) or diet supplemented with nucleotides (NT diet) for a period of 21 days. Blood samples, intestinal tissues and digesta were collected at necropsy and analyzed for morphology, digestive enzyme activities, microbial populations, peripheral immune cells, expression of intestinal innate immunity and barrier-related genes and proteins. Compared with NBW piglets, IUGR piglets had significantly lower average daily dry matter intake and body weight gain (*P*<0.05). Moreover, IUGR markedly decreased the villous height and villi: crypt ratio in duodenum (*P*<0.05), as well as the maltase activity in jejunum (*P*<0.05). In addition, IUGR significantly decreased the serum concentrations of IgA, IL-1βand IL-10 (*P*<0.05), as well as the percentage of peripheral lymphocytes (*P*<0.05). Meanwhile, the down-regulation of innate immunity-related genes such as TOLLIP (*P*<0.05), TLR-9 (*P* = 0.08) and TLR-2 (*P* = 0.07) was observed in the ileum of IUGR relative to NBW piglets. Regardless of birth weight, however, feeding NT diet markedly decreased (*P*<0.05) feed conversion ratio, increased the villous height in duodenum (*P*<0.05), activities of lactase and maltase in jejunum (*P*<0.05), count of peripheral leukocytes (*P*<0.05), serum concentrations of IgA and IL-1β as well as gene expressions of TLR-9, TLR-4 and TOLLIP in ileum (*P*<0.05). In addition, expressions of tight junction proteins (Claudin-1 and ZO-1) in ileum were markedly increased by feeding NT diet relative to CON diet (*P*<0.05). These results indicated that IUGR impaired growth performance, intestinal and immune function, but dietary nucleotides supplementation improved nutrients utilization, intestinal function and immunity.

## Introduction

Intra-uterine growth restriction (IUGR) refers to the impaired growth and development of a mammalian embryo/fetus or its organs during pregnancy [1, 2]. Approximately 5∼10% of human neonates suffer from IUGR [3]. Neonates with IUGR have increased morbidity and mortality during the early life period, including the delayed postnatal growth and development, as well as increased susceptibility to infection [4]. Studies have shown that functions of internal organs, nutrient metabolism and immune system are impaired in IUGR human-beings [5–8] and animal models [9–11].

The various nutritional interventions have been developed to optimize the growth and health of IUGR neonates [12–14]. Nucleotides are a group of bioactive agents playing important roles in nearly all biochemical processes, such as transferring chemical energy, biosynthetic pathways and coenzyme components [15]. The nucleotide requirements could be met by three sources including de novo synthesis, salvage pathways and food. Generally the milk of mammal animals have higher contents than any other food origin [16]. Under certain conditions such as stress, immunological challenges and disease state, exogenous nucleotides become essential nutrients to optimize intestinal and immunological function [15, 17, 18]. It has been demonstrated that nucleotide supplementation could increase weight gain and antibody responses to tetanus toxoid of infants [19, 20]. To our knowledge, however, it is lacking about the effects of nucleotide supplementation in formula on growth and health parameters of IUGR neonates, which are often fed artificial formula to ensure catch-up growth and development[21, 22]. Therefore, this study was to determine whether dietary supplementation of nucleotides could improve the growth performance, intestinal development and immune function of IUGR neonates. Piglets are generally accepted as the animal model for infant nutrition, because the structural and physiological similarities of gastrointestinal tract between pigs and human beings [23, 24]. As a multi-fetal domestic animal, moreover, pigs have exhibited naturally occurred IUGR due to utero-placental insufficiency [23].

## Materials and Methods

### Milk replacer diets

Milk replacer powder was formulated according to the previous study [25]. The nucleotide-supplemented diet was prepared by adding milk replacer powder with a mixture of pure nucleotides (29.6 g 5´-adenine-monophosphate, 14.2 g 5´-cytosine-monophosphate, 40.8 g 5´-guanosine-monophosphate, 5.8 g 5´-inosine-monophosphate, and 650.5 g 5´-uridine-monophosphate), a total of 740.9 g nucleotides per 100 kg milk replacer powder. Pure nucleotides were donated by Zhen-AO Group Co. Ltd. (Dalian, China) and had purities of 97%, according to the analysis of the manufacturer. The content of individual nucleotide in the final solution was designed according to the average content of nucleotides in sow milk during day 7 to day 28 after birth [26]. The formula milk was prepared by mixing 1 kg of milk replacer powder (dry matter, DM 87.5%) with 4 liters of water, in which nutrients composition and levels were similar as sow milk [9].

### Animal and treatment

All of the procedures were approved by the Institutional Animal Care and Use Committee of Sichuan Agricultural University.

According to the previous studies [25, 27], fourteen healthy pregnant sows at parity 3 gave birth at full term (115±2 d gestation). Newborn male piglets (Pig Improvement Company 327 × 1050) with birth weight near the mean birth weight (±0.5 standard deviation, SD) were identified as normal birth weight (NBW), whereas piglets at least 1.5 SD lower birth weight were defined as IUGR. Following this criteria, fourteen pairs of NBW piglets at 1.56 (SD 0.05) kg and IUGR littermates at 0.91 (SD 0.03) kg were selected from the 14 sows, who had same litter size (10 live piglets per litter). All piglets were weaned at 7 day of age and moved to be individually fed with milk-based diet every 3 hours by bottle feeding between 06.00 and 24.00 hours in nursing cages (0.8 m × 0.7 m × 0.4 m). For nutritional treatments, seven pairs of NBW and IUGR piglets were assigned to receive control diet (CON), while the other 7 pairs were allocated to receive nucleotides-supplemented diet (NT). Therefore, four groups of piglets were created and studied: NBW-CON; IUGR-CON; NBW-NT; IUGR-NT (n = 7). As the previous study [25], the milk-based diet was prepared by mixing 1 kg of formula powder with 4 litres of water to a milk solution, one hundred milliliter of milk-based diet contained 5.06g protein, 4.64g lactose and 5.20g lipids, which were similar as that in the same volume of sow milk, containing 5.00g protein, 5.06g lactose and 7.90g lipids [28]. All piglets had free access to drinking water. Room temperature was maintained at approximately 30°C and the humidity was controlled between 50% and 60%. The body weight (BW) and formula milk intake of piglets were recorded daily. The average daily dry matter intake (ADMI) was calculated via multiplying the average daily intake of formula milk by its DM content (%), while daily intake of formula milk was calculated as the difference between the offered amounts and the refusals.

### Blood sample collection

On day 28, D-xylose was orally administrated to piglets at the dose of 500 mg/kg BW after an overnight fast [29]. D-xylose solution was prepared by dissolving D-xylose powder (Sigma-Aldrich, St. Louis, MO, USA) at 50 mg/ml of deionized water. One hour after administration of the D-xylose solution, a 10-mL blood sample was collected by venipuncture of jugular vein. A part of the blood sample was injected into vacuum tubes containing sodium heparin for the examination of leucocytes and lymphocyte subtype, another part of the sample was allowed to coagulate for 40 min before centrifugation (10 min, 2,375 × g at 4°C), the plasma samples were stored at -80°C until analysis.

### Tissue sample collection

After blood sampling, all piglets were anaesthetized with an intravenous injection of pentobarbital sodium (50 mg/kg BW) and slaughtered. Piglets were weighed and crown-rump length (CRL) was taken (the supine length of the piglet from the crown of its head to the base of its tail) at d 28. Body mass index (BMI; BW/CRL^2^) was calculated for each piglet. The liver, spleen, kidney, heart and pancreas of each piglet were weighed immediately. The length and weight of the small intestine were measured after the removal of luminal contents. Duodenal, jejunal and ileal samples of approximately 2 cm in length were stored in 4% paraformaldehyde solution for histological analyses. The rest of the jejunum and ileum were snap frozen and stored at fridge with −80°C until further analysis. Finally, colonic digesta were collected immediately after removal of the colon and frozen at −80°C.

### Peripheral leucocytes and lymphocyte subtype detection

The examination of leucocytes (neutrophil, lymphocyte and monocyte counts) was conducted through an automatic blood analyser (Bayer HealthCare, Tarrytown, NY). Total peripheral blood lymphocytes were separated from heparinised peripheral blood, then stained with mouse anti-porcine CD3e-SPRD (PE-Cy5) (catalogue no. 4510–13), CD4a-FITC (catalogue no. 4515–02) and CD8a-PE (catalogue no. 4520–09), which were purchased from Southern Biotechnology Associates (Birmingham, AL, UK). PBS and 1.0% BSA (MP Biomedicals, Aurora, OH, USA) were used as diluents and washing buffer. Flow cytometry analysis was performed on a BD FACSCalibur flow cytometer (Becton Dickinson, San Jose, CA, USA), repeated for the same sample and compared for repeatability.

### Measurement of plasma Immunoglobulin Subset and Cytokines

Commercially available enzyme immunoassays were performed according to the instructions from the manufacturer for the following markers: IgA (Bethyl Lab. Inc., Montgomery, USA),IL-1β (R&D Systems, Oxford, UK), TNF-α (R&D Systems, Oxford, UK), IL-10 (Bio Source/Med Probe, Camarillo, CA, USA). Absorbance at 450 nm was determined using a Bio-Tek synergy HT microplate reader (BioTek Instruments, Inc., Winooski, USA). The detection limits were 12.5 ng/ml for IgA, 7.0 pg/ml for TNF-α, 30.0 pg/ml for IL-1β and 8.0 pg/ml for IL-10, respectively. The inter- and intra-assay coefficients of variation were less than 10%.

### Determination of D-xylose in plasma

The D-xylose absorption test was carried out according to the method described by Mansoori et al. (2009) [29]. Briefly, D-xylose standard solutions were prepared by dissolving D-xylose in saturated benzoic acid at concentrations of 0, 0.7, 1.3, 2.6 mmol/L, then D-xylose standard solutions and 50 μL of plasma were added to 5 mL of phloroglucinol color reagent solution (Sigma Chemical Inc., St. Louis, MO, USA) and heated at 100°C for 4 min. The samples were allowed to cool until room temperature in a water bath. The absorbance of all samples and standard solutions were measured using the spectrophotometer at 554 nm (Model 6100, Jenway LTD., Felsted, Dunmow, CM6 3LB, Essex, England, UK).The standard solution of 0 mmol/L D-xylose was considered as blank.

### Small-intestinal morphology

The duodenal, jejunal and ileal samples were preserved in 4% paraformaldehyde solution, then embedded in paraffin. Each sample (duodenum, jejunum and ileum) was used to prepare 5 slides and each slide had 3 sections (5 μm thickness), which were stained with eosin and hematoxylin. For each section, 20 well-oriented villi and crypts were measured for intestinal morphology (Optimus software version 6.5; Media Cybergenetics, North Reading, MA), then villi-crypt ratio (VCR) was calculated.

### Digestive enzyme activities

After thawing, the frozen jejunal sample (approximately 2 grams) was weighed and homogenized for 5 min in the 9 times volume of 50 mM Tris-HCl buffer (pH 7.0), then centrifuged at 3000g for 10 min. The supernatant was collected and stored at −20°C for the enzyme assay. Total proteins were extracted and the concentration was determined according to the procedure of bicinchoninic acid assay (Solarbio, Inc., Beijing, China). Activities of disaccharidaseincluding maltase, sucrose and lactase were measured using commercial kits according to the manufacturer’s instructions (Nanjing Jiancheng Bioengineering Institute, Nanjing, USA). The absorbance at 505 nm was determined with spectrophotometer (Beckman Coulter DU-800, CA, USA). The activities of disaccharidase were expressed as U/mg protein. One unit (U) was defined as 1 nmol of maltose, sucrose and lactose as substrate for the enzymatic reaction.

### Cell Cycle of Spleen by Flow Cytometry Method

The percentage of cells entering S and G2/M-phases of cell cycle were assessed by the flow cytometric analysis. At day 28 of the experiment, the spleen was excised from each piglet to determine the cell cycle stages by flow cytometry. Splenic cell suspension was prepared by dissecting spleen into small pieces and filtering them through the 300-mesh nylon gauze. Then, the cells were washed and suspended in phosphate buffer at a concentration of 1×10^6^ cells/mL. A total of 500 μL cell suspension was transferred into 5-mL culture tube and centrifuged at 3000g for 5 min. The cell suspension was perrmeabilized with 1 mL of 0.25% Triton X-100 for 20 min at 4°C, then the cells were washed with phosphate buffer. Propidium iodide (5 μL) was added into 100-μL cell suspension and incubated for 30 min at 4°C in the dark room. Finally, 400 μL of phosphate buffer was added and the cell cycle stages were assayed by flow cytometry (Becton Dickinson, San Jose, CA, USA) within 45 min and analyzed by ModFit software (Verity Software House, Inc., USA). Proliferating index value (PI value) was calculated following the formula:

$$PI=\frac{S+(G2+M)}{\left(G0/G1)+S+(G2+M\right)}\times 100\%$$

### Total RNA extraction and real-time PCR

Total RNA was extracted from the frozen intestinal tissues (approximately 100 mg) using Trizol Reagent (Invitrogen, Carlsbad, CA, USA), according to the manufacturer’s instructions. RNA integrity and quality were determined by agarose gel electrophoresis (1%) and spectrometry (A260/A280). RNA concentration was confirmed by nucleic-acid/protein analyzer (Beckman DU-800, CA, USA). A commercial reverse transcription (RT) kit (TaKaRa, Japan) was used for the synthesis of cDNA. The RT products (cDNA) were stored at –20°C for relative quantification by polymerase chain reaction (PCR). Primers were designed by Primer Express 3.0 (Applied Biosystems, Foster City, CA, USA) and shown in Table 1. cDNA was amplified using a real-time PCR system (ABI 7900HT, Applied Biosystems, USA). The mixture (10 μL) contained 5 μL of SYBR Green Supermix (TaKaRa, Japan), 1 μL of cDNA, 0.4 μL of each primer (10 μM), 0.2 μL of ROX Reference Dye and 3 μL of ddH_2_O. The cycling conditions were used as follows: denaturation at 95°C for 15 sec, followed by 40 cycles of denaturation at 95°C for 5 sec, annealing at 60°C for 30 sec, and extension step at 72°C for 15 sec. Product size was determined by agarose gel electrophoresis. The standard curve of each gene was run in duplicate and three times for obtaining reliable amplification efficiency values as described previously [30]. The correlation coefficients (r) of all the standard curves were > 0·99 and amplification efficiency values were between 90 and 110%. The most stable housekeeping genes (β-actin and GADPH) were chosen for normalization. Relative mRNA abundance was determined using the Δ cycle threshold (ΔCt) method, as outlined in the protocol of Applied Biosystems. In brief, a ΔCt value is the Ct difference between the target gene and the reference gene (ΔCt = Ct^target^− Ct^reference^). For each of the target genes, the ΔΔCt values of all the samples were calculated by subtracting the average ΔCt value of the corresponding IUGR-CON group. The ΔΔCt values were converted to fold differences by raising 2 to the power –ΔΔCt (2^–ΔΔCt^) according to Livak and Schmittgen (2001) [31].

**Table 1:** Primer sequences of the target and reference genes.^#^

Genes	Primer Sequence (5’-3’)	Product (bp)	GenBank accession
TLR-2	Forward: TCGAAAAGAGCCAGAAAACCAT	58	NM213761
	Reverse: CTTGCACCACTCGCTCTTCA		
TLR-4	Forward: AGAAAATATGGCAGAGGTGAAAGC	64	GQ304754
	Reverse: CTTCGTCCTGGCTGGAGTAGA		
TLR-9	Forward: AATCCAGTCGGAGATGTTTGCT	79	AY859728
	Reverse: GACCGCCTGGGAGATGCT		
MyD88	Forward: GTGCCGTCGGATGGTAGTG	65	NM001099923
	Reverse: TCTGGAAGTCACATTCCTTGCTT		
TRAF-6	Forward: GCTGCATCTATGGCATTTGAAG	70	AJ606305.1
	Reverse: CCACAGATAACATTTGCCAAAGG		
NF-κB	Forward: TGCTGGACCCAAGGACATG	60	AK348766.1
	Reverse: CTCCCTTCTGCAACAACACGTA		
IL-1β	Forward: TCTGCCCTGTACCCCAACTG	64	NM214055.1
	Reverse: CCAGGAAGACGGGCTTTTG		
IL-6	Forward: GATGCTTCCAATCTGGGTTCA	62	M80258.1
	Reverse: CACAAGACCGGTGGTGATTCT		
SIGIRR	Forward: ACCTGGGCTCCCGAAACTAC	62	AK239384.1
	Reverse: GTCATCTTCTGACACCAGGCAAT		
TOLLIP	Forward: CCCGCGCTGGAATAAGG	74	AK239879.1
	Reverse: CATCAAAGATCTCCAGGTAGAAGGA		
Claudin-1	Forward: TCTTAGTTGCCACAGCATGG	106	NM_001244539
	Reverse: CCAGTGAAGAGAGCCTGACC		
Occludin	Forward: TTCATTGCTGCATTGGTGAT	113	NM_001163647
	Reverse: ACCATCACACCCAGGATAGC		
ZO-1	Forward: CCGCCTCCTGAGTTTGATAG	97	AJ318101
	Reverse: CAGCTTTAGGCACTGTGCTG		
GLUT2	Forward: CCTGCTTGGTCTATCTGCTGTG	194	NM_001097417.1
	Reverse: TTGATGCTTCTTCCCTTTCTTT		
PEPT1	Forward: GATGAAATGTGAGCGTATGGG	109	AY180903.1
	Reverse: AAAGAGGGAGGATCTGGAAAA		
SGLT1	Forward: CCACTTTCCCTATAAAACCTCAC	151	NM_001164021.1
	Reverse: CTCCATCAAACTTCCATCCTCAG		
β-actin	Forward: GGCGCCCAGCACGAT	66	DQ845171.1
	Reverse: CCGATCCACACGGAGTACTTG		
GAPDH	Forward: TCGGAGTGAACGGATTTGGC	147	NM_001206359.1
	Reverse: TGCCGTGGGTGGAATCATAC		

^**#**^TLR, toll-like receptor; MyD88, myeloid differentiation factor 88; TRAF-6, tumor necrosis factor receptor-associated factor 6; NF-κB, nuclear factor kappa B; SIGIRR, single Ig IL-1-related receptor; IL: interleukin; TOLLIP, toll-interacting protein; GLUT2, glucose transporter 2; PEPT1, peptide transporter 1; SGLT1, Na^+^-dependent glucose transporter 1; GAPDH, glyceraldehyde-3-phosphate dehydrogenase; ZO-1, zonula occludens-1.

### Gut Microbial Population Determination

Bacterial DNA was extracted from the intestinal digesta using the Stool DNA Kit (Omega Bio-tek,Inc.,GA,USA) according to the manufacturer’s instructions. Quantitative RT-PCR for total bacteria was performed with SYBR Green PCR reagents (Takara, Kyoto, Japan), whereas quantitative RT-PCR for *Bifidobacterium*, *Lactobacillus*, *Bacillus* and *Escherichia coli* were performed with Taq Primers, fluorescent oligonucleotide probes were commercially synthesized (Life Technologies Ltd., Beijing, China). The RT-PCR primers and probes combination were presented in Table 2. A 10-fold serial dilution of the copies, ranging from 1 × 10^1^ to 1 × 10^12^ copies/μl, were used to construct the standard curves. The copy numbers (copies/μl) were calculated via measuring the concentration of the plasmid using the spectrophotometer (Beckman Coulter DU-800, CA, USA) according to the equation: DNA copy numbers = (DNA concentration in μg/μl × 6.0233 × 10^23^ copies/mol)/(DNA size (bp) × 660 × 10^6^). The standard curves of bacteria were generated by the cycle threshold (CT) values in each dilution that was measured in duplicate using a quantitative RT-PCR. Each standard curve was constructed by a linear regression of the plotted points, the CT values were plotted against the logarithm of the template copy numbers. From the slope of each standard curve, PCR amplification efficiency (E) was calculated following the equation that is E = 10^[−1/slope−1]^.

**Table 2:** Oligonucleotide primers and probes used for bacteriological analysis.

Primers / Probes	Sequence (5’-3’)		Tm(°C)	Product size(bp)
*Escherichia coli*	Forward:	CATGCCGCGTGTATGAAGAA	57	96
	Reverse:	CGGGTAACGTCAATGAGCAAA		
	Probe:	AGGTATTAACTTTACTCCCTTCCTC		
*Lactobacilli*	Forward:	GAGGCAGCAGTAGGGAATCTTC	55.7	126
	Reverse:	CAACAGTTACTCTGACACCCGTTCTTC		
	Probe:	AAGAAGGGTTTCGGCTCGTAAAACTCTGTT		
*Bifidobacterium*	Forward:	CGCGTCCGGTGTGAAAG	57	121
	Reverse:	CTTCCCGATATCTACACATTCCA		
	Probe:	ATTCCACCGTTACACCGGGAA		
*Bacillus*	Forward:	GCAACGAGCGCAACCCTTGA	57	92
	Reverse:	TCATCCCCACCTTCCTCCGGT		
	Probe:	CGGTTTGTCACCGGCAGTCACCT		
Total bacteria	Forward:	ACTCCTACGGGAGGCAGCAG	64.5	200
	Reverse:	ATTACCGCGGCTGCTGG		

### Western blotting

Protein extracts were obtained by homogenizing ileal tissues using the protein extraction kit (Beyotime Biotechnology, Jiangsu, China) according to the manufacturer’s guide. The protein content was measured using the bicinchoninic acid protein assay kit (Pierce, Rockford, IL, USA). The antibody was used in our experiment: goat polyclonal anti-ZO-1 (sc-8146, Santa Cruz Biotechnology, Santa Cruz, CA, USA), goat polyclonal anti-claudin-1 (sc-17658, Santa Cruz Biotechnology, Santa Cruz, CA, USA) and mouse monoclonal anti-β-actin (sc-47778, Santa Cruz Biotechnology, Santa Cruz, CA, USA). Western blot analysis was performed as previously described [32]. Chemiluminescence detection was performed using the ECL Plus^TM^ Western Blotting Detection System (Amersham, Arlington Heights, IL, USA) according to the manufacturer’s instructions. The relative expression of target protein was normalized using β-actin as the internal protein, the normalized values were used for comparison between groups.

### Statistical analysis

The data were analyzed by Duncan’s multiple comparisons for the 2 × 2 factorial experimental design using the General Linear Model (GLM) procedure of SPSS statistical software (Ver.20.0 for Windows, SPSS, Chicago, IL, USA) in the following model: *y*_*ijk*_ = *μ* + *a*_*i*_ + *b*_*j*_ + (*ab*)_*ij*_ + *e*_*ijk*_ (_*i*_ = 1, 2, _*j*_ = 1, 2, _*k*_ = 1, 2,…,14), where *y*_*ijk*_ represents the dependent variable, *μ* is the mean, *a*_*i*_ is the effect of BW (IUGR, NBW), *b*_*j*_ is the effect of Diet (CON, NT), (*ab*)_*ij*_ is the interaction between BW and Diet, and *e*_*ijk*_ the error term. Results are presented as means with their standard errors (SEM). Differences were considered as significant when *P* < 0.05, and a tendency was recognized when *P*< 0.10.

## Results

### Growth performance

For the whole experimental period, IUGR piglets had lower average daily gain (ADG) and ADMI (P<0.05) than that of NBW piglets in either CON or NT diet group. Moreover, regardless of the diet, IUGR piglets had lower (−24%, *P*<0.05) average daily gain (ADG) and ADMI (−25%, *P*<0.05) compared with NBW piglets, respectively. Meanwhile, the initial BW, final BW and BW gain of IUGR piglets were lower (−25∼37%, *P*<0.05) than those of NBW neonates. In addition, for either IUGR or NBW piglets, there was similar ADMI between CON and NT diet. Regardless of BW, however, ADG tended to be higher in piglets receiving NT relative to CON diet, consequently, feed conversion ratio (FCR) was markedly decreased (−17%, *P*<0.05)(Table 3).

**Table 3:** Effect of dietary nucleotides supplementation on the growth performance of intra-uterine growth-restricted (IUGR) and normal-birth weight (NBW) neonates.^#^

	CON		NT		SEM	*P* value		
	IUGR	NBW	IUGR	NBW		BW	Diet	BW×Diet
Initial weight (kg)	1.70^a^	2.70^b^	1.70^a^	2.72^b^	0.17	<0.05	0.90	0.87
Final weight (kg)	5.20^a^	7.80^b^	5.91^a^	7.86^b^	0.74	<0.05	0.13	0.38
Net weight gain (kg)	3.50^a^	5.09^b^	4.21^a^	5.14^b^	0.64	<0.05	0.09	0.29
ADG (g/d)								
Days 7–14	113^a^	195^b^	151^a^	191^b^	47	<0.05	0.36	0.27
Days 14–21	112^a^	192^b^	140^a^^b^	188^b^	52	<0.05	0.54	0.43
Days 21–28	275^a^	323^a^^b^	310^a^^b^	355^b^	58	0.05	0.16	0.95
Days 7–28	167^a^	236^b^	200^a^	245^b^	43	<0.05	0.09	0.30
ADMI (g/d)								
Days 7–14	104^a^	163^b^	108^a^	156^b^	34	<0.05	0.89	0.67
Days 14–21	130^a^	190^b^	129^a^	164^b^	43	<0.05	0.46	0.46
Days 21–28	201^a^	248^b^	190^a^	239^b^	39	<0.05	0.52	0.95
Days 7–28	145^a^	200^b^	143^a^	186^b^	32	<0.05	0.53	0.64
FCR*								
Days 7–14	0.92	0.84	0.72	0.82	0.12	0.64	0.08	0.22
Days 14–21	1.16	0.99	0.92	0.87	0.14	0.07	<0.05	0.24
Days 21–28	0.73	0.77	0.63	0.67	0.17	0.88	0.08	0.58
Days 7–28	0.87^b^	0.85^b^	0.71^a^	0.76^a^	0.09	0.72	<0.05	0.17

^**#**^Mean values with their standard errors, n = 7 in each group. BW, body weight; CON, control diet; NT, nucleotides-supplemented diet; ADG, average daily gain; ADMI, average daily DM intake; FCR, feed conversion ratio.

^a,b^Mean values within a row with different superscript letters were significantly different (*P*< 0.05).

*FCR was calculated via dividing the ADMI by its corresponding ADG.

### Organ indices

As shown in Table 4, regardless of the diet, weights of internal organs such as heart, liver, spleen, kidney, pancreas and intestine were markedly decreased (−27∼34%, *P*<0.05) in IUGR relative to NBW piglets. Moreover, the crown-rump length (CRL) and body mass index (BMI) at d 28 were lower (−9∼11%, *P*<0.05) in IUGR than that of NBW piglets, respectively. However, the relative intestinal length to BW was significantly higher (+15%, *P*<0.05) in IUGR relative to NBW piglets. Regardless of BW, BMI tended to be increased (+8%, *P* = 0.08) in piglets fed NT diet compared with piglets fed CON diet.

**Table 4:** Effects of dietary nucleotides supplementation on the organ index of intra-uterine growth-restricted (IUGR) and normal-birth weight (NBW) neonates.^#^

	CON		NT		SEM	*P* value		
	IUGR	NBW	IUGR	NBW		BW	Diet	BW×Diet
Day 28 CRL (cm)	42^a^	48^b^	43^a^	48^b^	3	<0.05	0.59	0.71
Heart wt (g)	25^a^	38^b^	27^a^	37^b^	6	<0.05	0.88	0.62
Liver wt (g)	119^a^	188^b^	152^a^^,^^b^	187^b^	34	<0.05	0.24	0.21
Spleen wt (g)	8^a^	13^b^	9^a^	12^b^	2	<0.05	0.99	0.38
Kidney wt (g)	31^a^	43^b^	32^a^	43^b^	6	<0.05	0.98	0.89
Pancreas wt (g)	10^a^	13^b^	9^a^	16^b^	2	<0.05	0.24	0.26
Intestinal wt (g)	280^a^	434^b^	329^a^	419^b^	68	<0.05	0.50	0.24
Intestinal L (cm)	832^a^	1039^b^	874^a^	1021^b^	116	<0.05	0.78	0.52
Intestinal L: wt (cm/g)	3.13^b^	2.41^a^	2.81^a^^,^^b^	2.47^a^^,^^b^	0.53	<0.05	0.56	0.40
Intestinal wt: BW (%)	5.78	5.71	5.54	5.33	7.9	0.66	0.35	0.83
Intestinal L: BW (cm/kg)	159.50^b^	138.32^a^^,^^b^	150.33^a^^,^^b^	130.64^a^	23.43	<0.05	0.26	0.75
Heart wt: BW (%)	0.48	0.49	0.45	0.47	0.05	0.58	0.22	0.74
Liver wt: BW (%)	2.27	2.44	2.51	2.39	0.30	0.84	0.45	0.23
Spleen wt: BW (%)	0.15	0.17	0.15	0.15	0.02	0.21	0.41	0.58
Kidney wt: BW (%)	0.60	0.56	0.54	0.54	0.07	0.61	0.18	0.46
Pancreas wt: BW (%)	0.18	0.17	0.16	0.20	0.03	0.34	0.77	0.16
Day 28 BMI (kg/m^2^)	29.26^a^	33.30^a^^,^^b^	32.61^a^^,^^b^	34.93^b^	3.58	<0.05	0.08	0.54

^**#**^Mean values with their standard errors, n = 7 in each group. BW, body weight; CON, control diet; NT, nucleotides-supplemented diet; wt, weight; L, length; CRL, crown-rump length; BMI, body mass index.

^a,b^Mean values within a row with different superscript letters were significantly different (*P* < 0.05).

### Intestinal morphology

IUGR significantly decreased the villous height and VCR (−11∼14%, *P*<0.05) of duodenum compared with NBW piglets. Irrespective of the BW, villous height in duodenum was markedly increased (+9%, *P*<0.05) in piglets fed NT diet relative to CON diet (Table 5).

**Table 5:** Effects of dietary nucleotides supplementation on the intestinal morphology of intra-uterine growth-restricted (IUGR) and normal-birth weight (NBW) neonates.^#^

	CON		NT		SEM	*P* value		
	IUGR	NBW	IUGR	NBW		BW	Diet	BW×Diet
Villous height (μm)								
Duodenum	466^a^	531^a^^,^^b^	517^a^^,^^b^	574^b^	65	<0.05	<0.05	0.87
Jejunum	480	523	481	513	50	0.06	0.81	0.76
Ileum	448	489	480	501	47	0.08	0.21	0.56
Crypt depth (μm)								
Duodenum	219	214	234	223	23	0.40	0.19	0.76
Jejunum	182	165	162	178	41	0.99	0.82	0.31
Ileum	184	168	183	160	35	0.16	0.76	0.78
VCR								
Duodenum	2.15^a^	2.50^a^^,^^b^	2.23^a^^,^^b^	2.60^b^	0.39	<0.05	0.57	0.93
Jejunum	2.75	3.37	3.06	3.03	0.71	0.30	0.96	0.24
Ileum	2.53	2.99	2.77	3.23	0.68	0.08	0.35	0.98

^**#**^Mean values with their standard errors, n = 7 in each group. CON, control diet; NT, nucleotides-supplemented diet; BW, body weight.

^a,b^Mean values within a row with different superscript letters were significantly different (*P*< 0.05).

### Digestive enzyme activities

IUGR piglets had markedly decreased maltase activity (−24%, *P*<0.05) compared with NBW piglets. Regardless of BW, however, piglets fed NT diet had increased activities of lactase and maltase (+35∼45%, *P*<0.050), also a tendency to increase sucrase activity (+34%, *P* = 0.06) (Table 6).

**Table 6:** Effects of dietary nucleotides supplementation on enzyme activities in the jejunum of intra-uterine growth-restricted (IUGR) and normal-birth weight (NBW) neonates.^#^

	CON		NT		SEM	*P* value		
	IUGR	NBW	IUGR	NBW		BW	Diet	BW×Diet
Lactase (U/mg protein)	20.01^a^^,^^b^	13.98^a^	26.37^b^	22.94^b^	6.22	0.06	<0.05	0.59
Maltase (U/mg protein)	45.69^a^	66.01^a^^,^^b^	67.90^a^^,^^b^	82.59^b^	20.24	<0.05	<0.05	0.72
Sucrase (U/mg protein)	11.96	14.61	17.15	18.47	5.51	0.38	0.06	0.77

^**#**^Mean values with their standard errors, n = 7 in each group. CON, control diet; NT, nucleotides-supplemented diet; BW, body weight

^a,b^Mean values within a row with different superscript letters were significantly different (*P*< 0.05).

### Concentration of plasma D-xylose

**Fig 1:**
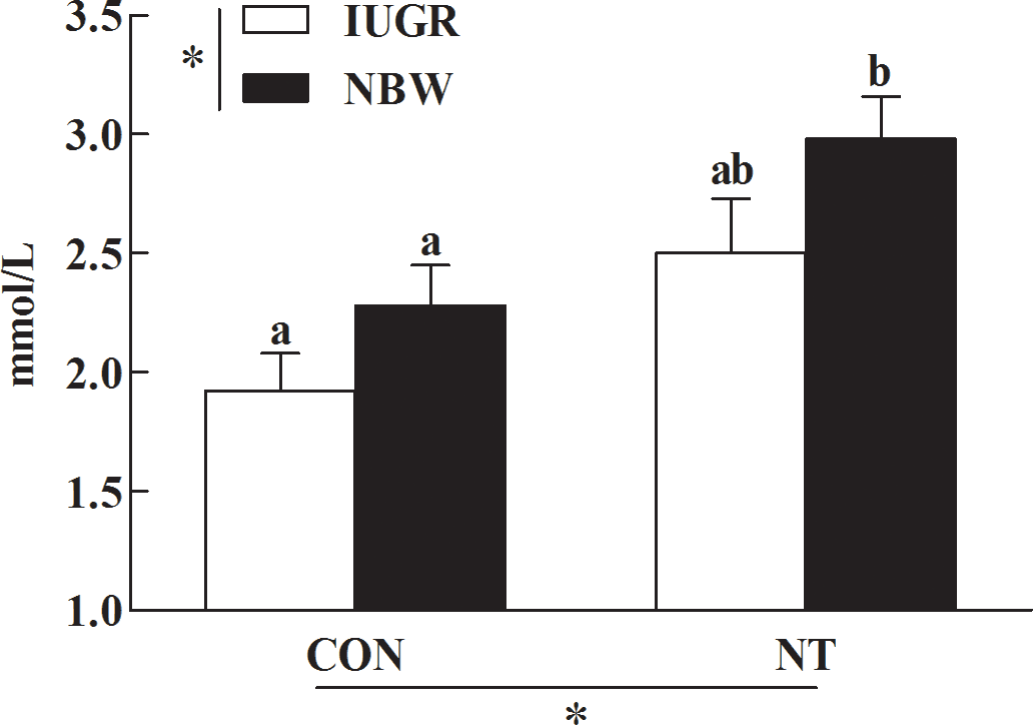
Effect of dietary nucleotides supplementation on the plasma concentration of D-xylose of IUGR and NBW piglets. Values are means, with standard errors represented by vertical bars, *n* = 7 in each group. IUGR, intrauterine growth restriction; NBW, normal-birth weight; CON, control diet; NT, nucleotides-supplemented diet; ZO-1, zonula occludens protein-1; ^a,b^ Mean values with unlike letters were significantly different (*P*< 0.05). * *P*< 0.05 for the respective sources of variation.

The plasma concentration of D-xylose was markedly lower (−16%, *P*<0.05) in IUGR relative to NBW piglets (Fig 1). However, feeding NT diet significantly increased (+30%, *P*<0.05) the plasma concentration of D-xylose in piglets.

### Plasma cytokines and immunoglobulin A

As shown in Table 7, IUGR significantly decreased the plasma concentrations of IgA (−16%, *P*<0.05), IL-1β (−11%, *P*<0.05) and IL-10 (−9%, *P*<0.05), as well as the ratio of IL-1β: IL-10 (−10%, *P*<0.05). Regardless of BW, the plasma concentrations of IgA (+16%, *P*<0.05) and IL-1β (+12%, *P*<0.05) as well as the ratio of IL-1β: IL-10were markedly increased (+23%, *P*<0.05) by feeding NT diet compared with CON diet. Meanwhile, the plasma concentration of IL-1β in IUGR piglets fed NT diet was normalized to be similar as that in NBW piglets (*P*<0.05).

**Table 7:** Effects of dietary nucleotides supplementation on the plasma concentrations of immunoglobulin A and cytokines of intra-uterine growth-restricted (IUGR) and normal-birth weight (NBW) neonates.^#^

	CON		NT		SEM	*P* value		
	IUGR	NBW	IUGR	NBW		BW	Diet	BW×Diet
IgA (ng/ml)	43.79^a^	54.42^b^	53.41^b^	61.00^c^	8.31	<0.05	<0.05	0.49
TNF-α (pg/ml)	143.55	147.31	153.00	148.50	19.17	0.96	0.49	0.59
IL-1β (pg/ml)	232.84^a^	280.46^b^	279.68^b^	294.44^b^	38.39	<0.05	<0.05	0.19
IL-10 (pg/ml)	73.42^a^	79.20^a^^,^^b^	72.74^a^	80.89^b^	6.99	<0.05	0.84	0.62
TNF-α:IL-10	1.79	1.95	2.04	2.04	0.35	0.22	0.55	0.54
IL-1β:IL-10	2.88^a^	3.54^b^	3.90^b^	4.02^b^	0.62	<0.05	<0.05	0.15

^**#**^Mean values with their standard errors, n = 7 in each group. CON, control diet; NT, nucleotides-supplemented diet; BW, body weight; IgA, immunoglobulin A; TNF-α, tumour necrosis factor-alpha; IL, interleukin.

^a,b,c^Mean values within a row with different superscript letters were significantly different (*P*< 0.05).

### Composition of peripheral leucocytes and lymphocyte percentages

As shown in Table 8, IUGR significantly increased the count (+138%, *P*<0.05) and percentage (+117%, *P*<0.05) of neutrophils, but markedly decreased (−12%, *P*<0.05) the percentage of lymphocytes. Regardless of BW, the count of leukocytes was higher (+34%, *P*<0.05) in piglets fed NT diet than that in piglets fed CON diet, moreover, the count of lymphocytes tended to increase (+33%, *P* = 0.07) in piglets fed NT diet. Furthermore, the percentage of G_2_M phase splenocytes was markedly lower in IUGR relative to NBW piglets (−46%, *P*<0.05) (Table 9).

**Table 8:** Effects of dietary nucleotides supplementation on count and percentage of blood leukocyte, neutrophil, lymphocyte, monocyte and percentage of T-cell subsets of intra-uterine growth-restricted (IUGR) and normal-birth weight (NBW) neonates.^#^

	CON		NT		SEM	*P* value		
	IUGR	NBW	IUGR	NBW		BW	Diet	BW×Diet
Leukocytes (10^9^/L)	16.68^a^	21.32^a^^,^^b^	28.35^b^	22.61^a^^,^^b^	8.09	0.85	<0.05	0.09
Neutrophils (10^9^/L)	3.96^a^^,^^b^	1.49^a^	4.98^b^	2.27^a^^,^^b^	1.23	<0.05	0.38	0.90
Lymphocytes (10^9^/L)	12.61^a^	19.49^a^^,^^b^	22.97^b^	19.84^a^^,^^b^	7.65	0.52	0.07	0.09
Monocytes (10^9^/L)	0.11	0.33	0.39	0.50	0.24	0.40	0.28	0.79
Neutrophils (%)	25.94^b^	7.01^a^	16.67^a^^,^^b^	12.63^a^	6.05	<0.05	0.67	0.10
Lymphocytes (%)	73.40^a^	91.41^b^	81.94^a^^,^^b^	85.82^a^^,^^b^	11.72	<0.05	0.73	0.10
Monocytes (%)	0.66	1.56	1.41	1.54	0.54	0.26	0.41	0.40
CD3^+^ (%)	60.96	59.33	58.65	59.75	6.92	0.92	0.73	0.62
CD4^+^ (%)	32.99	28.95	32.66	31.60	6.11	0.29	0.63	0.53
CD8^+^ (%)	26.31	20.83	21.81	24.21	5.59	0.48	0.79	0.08
CD4^+^ /CD8^+^	1.33	1.44	1.59	1.35	0.43	0.71	0.62	0.30

^**#**^Mean values with their standard errors, n = 7 in each group. CON, control diet; NT, nucleotides-supplemented diet; BW, body weight.

^a,b^Mean values within a row with different superscript letters were significantly different (*P*< 0.05).

**Table 9:** Effects of dietary nucleotides supplementation on cell cycle phase of spleen in intra-uterine growth-restricted (IUGR) and normal-birth weight (NBW) neonates.^#^

	CON		NT		SEM	*P* value		
	IUGR	NBW	IUGR	NBW		BW	Diet	BW×Diet
G_0_/G_1_	79.94	73.49	78.31	76.93	8.80	0.31	0.81	0.50
G_2_+M	4.75^a^	9.86^b^	3.94^a^	6.16^a^^,^^b^	1.94	<0.05	0.12	0.31
S	15.31	16.65	17.56	16.91	4.28	0.93	0.74	0.79
PI value	20.06	26.51	21.50	23.07	4.38	0.29	0.79	0.52

^**#**^Mean values with their standard errors, n = 7 in each group. CON, control diet; NT, nucleotides-supplemented diet; BW, body weight; PI, Proliferating index.

^a,b^Mean values within a row with different superscript letters were significantly different (*P*< 0.05).

### Gut Microbial Population

IUGR piglets had markedly lower (−4%, *P*<0.05) population of *Bacillus* in colonicdigestacomparedwith NBW piglets, but no signficant differences were observed for pouplations of *Escherichia coli*, *Bifidobacterium*,*Lactobacilli* and total bacteria among groups (Table 10).

**Table 10:** Effects of dietary nucleotides supplementation on the selected microbial populations (log10 copies/g of wet digesta) in colonic digesta of intrauterine growth restricted (IUGR) and normal-birth weight (NBW) neonates.^#^

	CON		NT		SEM	*P* value		
	IUGR	NBW	IUGR	NBW		BW	Diet	BW×Diet
Total bacteria	10.35	10.27	10.41	10.29	0.30	0.41	0.76	0.89
*Escherichia coli*	6.92	6.72	6.79	6.44	0.46	0.15	0.27	0.70
*Lactobacillus*	7.16	7.23	7.31	7.45	0.66	0.71	0.50	0.89
*Bifidobacterium*	6.00	5.98	6.07	6.05	0.31	0.82	0.97	0.99
*Bacillus*	7.10	7.35	6.98	7.34	0.32	<0.05	0.60	0.67

^**#**^Mean values with their standard errors, n = 7 in each group. CON, control diet; NT, nucleotides-supplemented diet; BW, body weight.

### Gene and protein expression in the ileum

**Fig 2:**
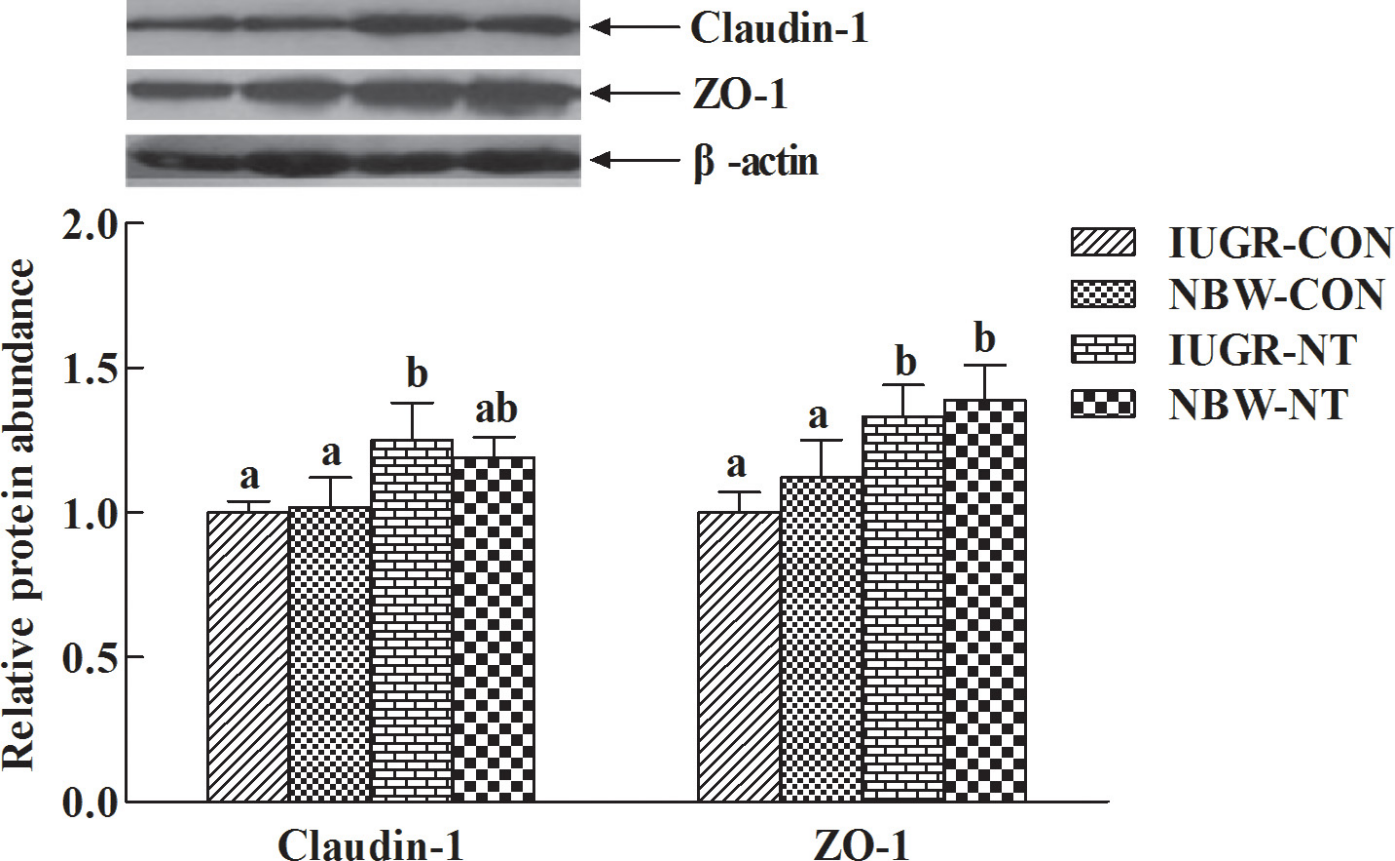
Effects of dietary nucleotides supplementation on protein expressions of Claudin-1 and ZO-1 in the ileum of IUGR and NBW piglets. Values are means, with standard errors represented by vertical bars, *n* = 7 in each group. The value of protein expression means densitometry units of selected protein/densitometry units of β-actin detected by Western blotting. IUGR, intrauterine growth restriction; NBW, normal-birth weight; CON, control diet; NT, nucleotides-supplemented diet; ZO-1, zonula occludens protein-1. ^a,b^Mean values with unlike letters were significantly different (*P*< 0.05).

As shown in Table 11, IUGR markedly decreased (−15%, *P*<0.05) gene expression of TOLLIP, but it was markedly increased (+22%, *P*<0.05) in piglets fed NT diet relative to CON diet. Meanwhile, IUGR tended to decrease gene expressions of TLR-9 (−26%, *P* = 0.08) and TLR-2 (−22%, *P* = 0.07), but feeding NT diet significantly increased (+25∼58%, *P*<0.05) gene expressions of TLR-9, TLR-4 and Claudin-1. In addition, gene expressions of GLUT2 (+22%, *P* = 0.07) and PEPT1 (+41%, *P*<0.05) were increased by feeding NT diet relative to CON diet. No significant differences were observed between IUGR and NBW piglets, but feeding NT diet markedly increased (+21∼28%, *P*<0.05) protein expressions of Claudin-1 and ZO-1 compared with CON diet (Fig 2).

**Table 11:** Effects of dietary nucleotides supplementation on mRNA abundance of innate immune related genes in ileum of intra-uterine growth-restricted (IUGR) and normal-birth weight (NBW) neonates.^#^

	CON		NT		SEM	*P* value		
	IUGR	NBW	IUGR	NBW		BW	Diet	BW×Diet
MyD88	1.00	1.07	1.05	0.97	0.17	0.93	0.72	0.27
TLR-9	1.00^a^	1.26^a^	1.48^a^^,^^b^	2.08^b^	0.58	0.08	<0.05	0.49
TLR-2	1.00^a^	1.30^a^^,^^b^	1.28^a^^,^^b^	1.63^b^	0.42	0.07	0.08	0.88
TRAF-6	1.00	0.95	1.08	0.92	0.18	0.17	0.80	0.43
TLR-4	1.00^a^	1.05^a^	1.44^b^	1.12^a^	0.27	0.19	<0.05	0.14
IL-6	1.00	1.14	0.97	1.26	0.34	0.13	0.78	0.59
NF-κB	1.00	0.89	0.96	1.08	0.17	0.89	0.31	0.11
SIGIRR	1.00	0.88	0.84	1.03	0.21	0.67	0.92	0.07
IL-1β	1.00	1.12	1.03	0.85	0.28	0.93	0.40	0.27
TOLLIP	1.00^a^	1.11^a^	1.15^a^	1.43^b^	0.17	<0.05	<0.05	0.27
ZO-1	1.00^a^^,^^b^	0.88^a^	1.23^b^	1.07^a^^,^^b^	0.24	0.19	0.05	0.86
Occludin	1.00	1.02	1.07	0.94	0.26	0.63	0.80	0.54
Claudin-1	1.00^a^	1.19^a^^,^^b^	1.35^a^^,^^b^	1.4^b^	0.30	0.32	<0.05	0.62
GLUT2	1.00^a^	1.16^a^^,^^b^	1.38^b^	1.26^b^	0.28	0.47	0.07	0.53
PEPT1	1.00^a^	0.99^a^	1.35^b^	1.45^b^	0.20	0.82	<0.05	0.85
SGLT1	1.00	0.98	1.14	1.05	0.17	0.75	0.51	0.80

^**#**^Mean values with their standard errors, n = 7 in each group. CON, control diet; NT, nucleotides-supplemented diet; BW, body weight; TLR, toll-like receptor; MyD88, myeloid differentiation factor 88; TRAF-6, tumor necrosis factor receptor-associated factor 6; NF-κB, nuclear factor kappa B; SIGIRR, single Ig IL-1-related receptor; IL: interleukin; TOLLIP, toll-interacting protein; GLUT2, glucose transporter 2; PEPT1, peptide transporter 1; SGLT1, Na^+^-dependent glucose transporter 1.

^a,b^Mean values within a row with different superscript letters were significantly different (*P*< 0.05).

## Discussion

The previous studies have shown that IUGR delayed postnatal growth [9, 25]. It has been proposed that the impaired intestinal function [33], endocrine status [34] and nutrient metabolism [35, 36] contributes to the growth check of IUGR neonates. In this study, there was lower formula milk intake and growth rate in IUGR relative to NBW piglets, however, supplementing nucleotides in formula markedly improved nutrients utilization, as indicated by the decreased FCR. Accordingly, the growth rate was faster in piglets fed NT relative to CON diet. Consistently, Singhal et al. (2010) demonstrated that feeding nucleotide-supplemented formula increased body weight gain of infants [19], however, some other studies in pig model showed that NT diet did not markedly affect body weight gain and FCR [37, 38]. The growth response of neonates to NT diet could be related to the physiological stage, supplemental contents and type of nucleotides [20, 37, 39]. In this study, nucleotides supplemented in formula had similar pattern and contents as the 5´-monophosphate nucleotide in sow milk, which is higher than that included in weaning diet for piglets [37, 38].

In order to clarify the mechanism of growth-promoting effect in neonates by supplementing nucleotides, the intestinal responses of IUGR piglets to NT diet were further investigated. Consistent with previous results [27, 40], IUGR impaired intestinal morphology and digestive enzyme activities, which are important factors to delay the postnatal growth. However, feeding NT diet increased the villous height, activities of lactase and maltase, thus resulting in the better feed conversion ratio relative to piglets fed CON diet. The improvements on intestinal morphology and enzyme activity could be related to the nucleotides, which are demanded to enhance the proliferation and maturation of enterocytes [41, 42]. In addition, as reported before [43, 44], the decreasing plasma concentration of D-xylose in IUGR piglets suggests the poor absorptive capability of IUGR intestine. In contrast, feeding NT diet markedly increased the plasma concentration of D-xylose and intestinal gene expression of PEPT1 in both IUGR and NBW piglets, suggesting the nutrients absorption may be increased by feeding NT diet.

In this study, moreover, the immunological response of IUGR piglets to NT diet was determined. There was decreasing plasma concentration of IgA in IUGR relative to NBW piglets. Consistently, the cord blood levels of IgG, IgA and IgM were markedly lower in IUGR relative to normal infants [45]. However, an one year longitudinal study showed no significant differences were observed for plasma levels of IgG, IgA and IgM between IUGR and NBW infants [46]. Regardless of BW, however, feeding NT diet markedly increased plasma concentration of IgA, which is also demonstrated to be higher in weaned pigs receiving nucleotides-containing ingredients [37, 47]. Similarly, the infants receiving nucleotides-enriched formula had increased IgG antibody concentrations to tetanus and diphtheria toxoid vaccines [20]. In addition, cellular immunity may be impaired in IUGR piglets, as indicated by the lower lymphocyte percentage, decreased concentrations of IL-1β and IL-10 in IUGR relative to NBW piglets. Furthermore, IUGR may negatively affect spleen function according to the lower percentages of G_2_M phase splenocytes, which would inhibit the proliferation of immune cells in spleen [48, 49]. Accordingly, the counts of lymphocyte and T-cells were significantly lower in IUGR infants [46]. In this study, however, cellular immunity-related leukocyte counts and IL-1β concentration as well as IL-1β:IL-10 were markedly increased in IUGR piglets fed NT diet, suggesting IUGR neonates may preferentially utilize nucleotides for cellular immunity. It has been reported that the nucleotides-deprived pyrimidine and purine bases are highly required for leukocyte proliferation [50].

Although previous study indicated that NT supplementation is able to improve intestinal microbiota in infants [51], the effect of NT diet on microbial populations was not observed, except the lower population of Bacillus in IUGR relative to NBW piglets in the present study. Toll-like receptors are typical pattern recognition receptors in mediating mucosal innate host defense to maintain mucosal and commensal homeostasis [52]. The MyD88, TRAF-6 and NF-κB are downstream signaling molecules shared by TLR-2, 4 and 9 [53], while SIGIRR and TOLLIP are crucial regulators negatively regulating the NF-κB signaling response [54, 55]. It has been demonstrated that the TLR-4-Myd88-NF-κB signal pathway is involved in the inflammation [56]. In this study, the lower gene expression of TOLLIP in ileum suggests that IUGR intestine may have immature innate immunity. The lower expression of TOLLIP has been observed in necrotizing enterocolitis intestine with the excessive inflammatory response to colonizing bacteria [57]. Intriguingly, feeding NT diet markedly increased gene expressions of TLR-9, TLR-4 and TOLLIP, indicating the positive effect of nucleotides supplementation on intestinal innate immunity. The signaling extent of TLR-4-Myd88-NF-κB pathway has closely related to the intestinal barrier [58], which may be improved by supplementing nucleotides, as shown by the increased protein expression of Claudin-1 and ZO-1 in ileum of piglets fed NT diet. Claudin-1, ZO-1 and Occludin are typical structural proteins of epithelial tight junction [59], the higher expression of these proteins indicates there was decreasing risk of inflammatory bowel diseases [60].

In conclusion, IUGR piglets had impaired growth, intestinal and immune function relative to NBW piglets. However, dietary nucleotides supplementation improved feed efficiency, associated with better digestive and absorptive capability as well as immune function of IUGR piglets.
